# PPG neurons in the nucleus of the solitary tract modulate heart rate but do not mediate GLP-1 receptor agonist-induced tachycardia in mice

**DOI:** 10.1016/j.molmet.2020.101024

**Published:** 2020-05-21

**Authors:** Marie K. Holt, Daniel R. Cook, Daniel I. Brierley, James E. Richards, Frank Reimann, Alexander V. Gourine, Nephtali Marina, Stefan Trapp

**Affiliations:** 1 Centre for Cardiovascular and Metabolic Neuroscience, Department of Neuroscience, Physiology & Pharmacology, UCL, London, UK; 2 Wellcome Trust/MRC Institute of Metabolic Science (IMS), Addenbrookes Hospital, University of Cambridge, Cambridge, UK

**Keywords:** GLP-1, Cardiovascular function, PPG neurons, Biotelemetry, Sympathetic outflow, Chemogenetics

## Abstract

**Objective:**

Glucagon-like peptide-1 receptor agonists (GLP-1RAs) are used as anti-diabetic drugs and are approved for obesity treatment. However, GLP-1RAs also affect heart rate (HR) and arterial blood pressure (ABP) in rodents and humans. Although the activation of GLP-1 receptors (GLP-1R) is known to increase HR, the circuits recruited are unclear, and in particular, it is unknown whether GLP-1RAs activate preproglucagon (PPG) neurons, the brain source of GLP-1, to elicit these effects.

**Methods:**

We investigated the effect of GLP-1RAs on heart rate in anaesthetized adult mice. In a separate study, we manipulated the activity of nucleus tractus solitarius (NTS) PPG neurons (PPG^NTS^) in awake, freely behaving transgenic Glu-Cre mice implanted with biotelemetry probes and injected with AAV-DIO-hM3Dq:mCherry or AAV-mCherry-FLEX-DTA.

**Results:**

Systemic administration of the GLP-1RA Ex-4 increased resting HR in anaesthetized or conscious mice, but had no effect on ABP in conscious mice. This effect was abolished by β-adrenoceptor blockade with atenolol, but unaffected by the muscarinic antagonist atropine. Furthermore, Ex-4-induced tachycardia persisted when PPG^NTS^ neurons were ablated, and Ex-4 did not induce expression of the neuronal activity marker cFos in PPG^NTS^ neurons. PPG^NTS^ ablation or acute chemogenetic inhibition of these neurons via hM4Di receptors had no effect on resting HR. In contrast, chemogenetic activation of PPG^NTS^ neurons increased resting HR. Furthermore, the application of GLP-1 within the subarachnoid space of the middle thoracic spinal cord, a major projection target of PPG neurons, increased HR.

**Conclusions:**

These results demonstrate that both systemic application of Ex-4 or GLP-1 and chemogenetic activation of PPG^NTS^ neurons increases HR. Ex-4 increases the activity of cardiac sympathetic preganglionic neurons of the spinal cord without recruitment of PPG^NTS^ neurons, and thus likely recapitulates the physiological effects of PPG neuron activation. These neurons therefore do not play a significant role in controlling resting HR and ABP but are capable of inducing tachycardia and so are likely involved in cardiovascular responses to acute stress.

## Highlights

• Activation of PPG neurons triggers increases in heart rate in mice.
• PPG neurons do not provide a tonic sympathetic drive to the heart.
• The tachycardic effect of systemic Ex-4 is not mediated by PPG neurons.
• GLP-1 receptor activation has a sympathoexcitatory effect that increases heart rate.
• Local activation of GLP-1R in the spinal cord is sufficient to elicit tachycardia.

## Graphical abstract

**Figure:**
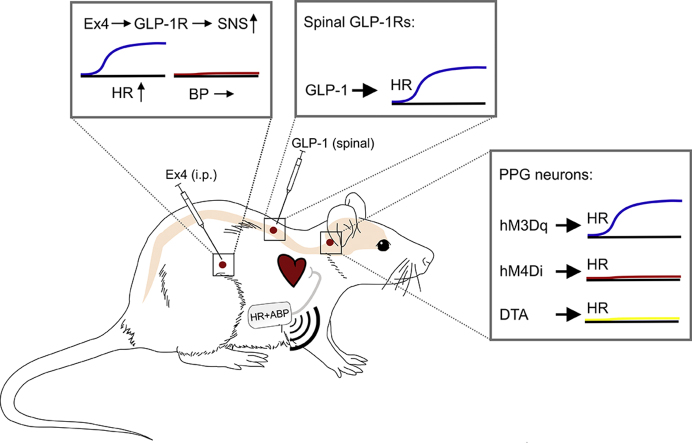

## 1 Introduction

Glucagon-like peptide-1 (GLP-1) is best known as an incretin that is released from the gut into the bloodstream postprandially and enhances insulin secretion. Based on that function, GLP-1 receptor agonists (GLP-1RAs), such as exendin-4 (Ex-4), are in clinical use to treat type 2 diabetes mellitus. However, both animal studies and clinical observations have established that GLP-1RAs have cardiovascular effects, including an increase in heart rate (HR) [[1], [2], [3], [4], [5], [6], [7]]. Despite the magnitude of these effects being typically modest, the high incidence of cardiovascular disease as a comorbidity of type 2 diabetes makes this observation pertinent and analysis of the underlying mechanisms worthwhile.

Arguably, it seems counterintuitive that a gut peptide involved in blood glucose control should be involved in cardiovascular control. However, GLP-1 is also produced within the brain, and there it is involved primarily in the regulation of food intake, but also in stress responses [[8], [9], [10], [11], [12], [13], [14], [15], [16]]. In fact, food intake, anxiety-like behaviour, corticosterone levels, and sympathetic activity are all modulated in response to challenges to survival, including acute stress [[17], [18], [19]] with GLP-1 being implicated in the modulation of all these functions. Within the brain, GLP-1 suppresses food and water consumption, decreases reward, drives anxiety-like behaviour, activates the hypothalamic-pituitary-adrenal (HPA) axis, and increases HR and arterial blood pressure (ABP) [[20], [21], [22], [23], [24]].

This raises numerous questions. First, where are the relevant populations of GLP-1 receptors (GLP-1Rs) for cardiovascular control located? Are these targets for brain-derived, rather than gut-derived, GLP-1? And if so, does that have consequences for the clinical use of current GLP-1RAs or for the use of future drugs designed to potentiate the activity of GLP-1-producing neurons in the brain? While it is now well established that both systemically and centrally applied GLP-1RAs can increase HR, the neural circuits involved remain controversial [5,6,22,[24], [25], [26], [27], [28]]. The parasympathetic [2,3,5,25] and sympathetic [1,13,24,29] nervous systems have both been implicated in these effects, but the contribution of endogenous brain-derived GLP-1 to the modulation of HR and ABP remains inconclusive. The only observation related to endogenous GLP-1 stems from Barragan et al. (1999), who found no effect of intracerebroventricular (i.c.v.) infusion of the GLP-1R antagonist, Ex-9, on HR and ABP [3]. More recently, Ghosal et al. demonstrated that the cardiovascular response to restraint stress was reduced in mice lacking GLP-1R in *Sim1* neurons in the paraventricular nucleus of the hypothalamus (PVN) [13], adding to a wealth of evidence pointing to a role for brain GLP-1 in central responses to stressful stimuli [[8], [9], [10], [11], [12], [13],^20^,[30], [31], [32], [33], [34]].

The main source of GLP-1 within the brain is preproglucagon (PPG) neurons of the lower brainstem [8]. The effect induced by i.c.v. delivery of GLP-1RAs likely recapitulates a physiological role of PPG neurons in cardiovascular control. In support of this, PPG neurons have been found to project to the pre-sympathetic nuclei of the PVN and the rostral ventrolateral medulla (RVLM) [35,36] as well as directly to sympathetic preganglionic neurons (SPN) located in the intermediolateral cell column (IML) and central autonomic area (CAA) of the spinal cord [37]. GLP-1Rs have been identified on both sympathetic neurons in the PVN, RVLM, and lamina X of the spinal cord, as well as in cardiac vagal preganglionic neurons of the nucleus ambiguous and dorsal motor nucleus of the vagus [[38], [39], [40]].

We recently showed that selective activation of PPG neurons in the nucleus tractus solitarius (PPG^NTS^) neurons with chemogenetic methods produces a suppression of food consumption and that their activity is necessary for stress-induced suppression of feeding [8]. Here we build on those findings by investigating the effect of systemic GLP-1R activation on HR, the involvement of PPG^NTS^ neurons in these effects, and the physiological role of PPG^NTS^ neurons in cardiovascular control. We demonstrate that, in the mouse, GLP-1R activation has no effect on resting ABP but elicits significant tachycardia, which is mediated by an increase in sympathetic outflow. Direct application of GLP-1 onto the thoracic spinal cord was sufficient to elicit robust increases in HR, and ablation of PPG^NTS^ neurons did not prevent tachycardia following systemic administration of GLP-1. Finally, we show through chemogenetic activation that PPG^NTS^ neurons have the capacity to increase HR, but also demonstrate through both chemogenetic inhibition and ablation that PPG^NTS^ neuronal activity do not provide tonic control of cardiac chronotropy under resting conditions.

## 2 Materials and methods

### 2.1 Animals

We used adult Glu-Cre [[41], [42], [43]] and Glu-YFP [44] mice of either sex on a C57Bl6 background. Mice were usually group-housed and kept on a 12-h dark/light cycle with water and chow available *ad libitum*. Experiments were conducted in accordance with the U.K. Animals (Scientific Procedures) Act, 1986, with appropriate ethical approval.

### 2.2 Anaesthetized preparations

Naïve 13- to 26-week-old Glu-YFP (or Glu-Cre, where they underwent previous stereotaxic viral transduction) mice were anaesthetized with urethane (1.3 g/kg, intraperitoneally (i.p), following 4% isoflurane induction) or urethane (650 mg/kg) + alpha-chloralose (50 mg/kg) intravenously. The trachea was cannulated with a plastic tube and core body temperature was kept at 37 °C throughout the experiment with a servo-controlled heating pad. The femoral vein was cannulated for the infusion of drugs. The depth of anaesthesia was monitored using stability of HR, corneal reflexes and absence of flexor responses to paw-pinch.

#### 2.2.1 Stereotaxic injections

All stereotaxic injections were performed on 9- to 22-week-old Glu-Cre mice. Animals were anaesthetized with a mixture of ketamine hydrochloride (50 mg/kg; intramuscular (i.m.)) and medetomidine (1 mg/kg; i.m.) or 1.5–2.5% isoflurane and the skull was fixed in a stereotaxic frame. A core temperature of 37 °C was maintained throughout the procedure using a heating mat. The nose of the mouse was pushed downwards, creating a right angle between the nose and the neck to expose part of the brainstem normally covered by the cerebellum. A longitudinal incision was made from the occipital bone to the first vertebra. Obex was exposed by parting overlying muscle layers and the atlanto-occipital membrane was pierced using a 30G needle. Adeno-associated viral vectors (AAV; Table 1) were injected bilaterally (250 nl) using the following coordinates from obex: 0.50 mm lateral; 0.10 mm rostral; 0.35 mm ventral. Anaesthesia was reversed with Antisedan (2.5 mg/kg; i.m.) and mice received buprenorphine (0.5 mg/kg; subcutaneous (s.c.)) for pain relief and 100 μl sterile saline (s.c.) for fluid support. The animals were left to recover in a 34 °C chamber until fully awake, and experiments commenced 2–4 weeks after viral gene transfer.

**Table 1:** Sources of virus and antibody preparations used.

Virus/Antibody	Application	Source	References
AAV8-DIO-hM_3_Dq:mCherry	Activation of Cre-expressing PPG neurons	VVF, ZNZ, Zurich	pAAV-hSyn-DIO-hM3D(Gq)-mCherry was a gift from Bryan Roth [67]
AAV8-mCherry-FLEX-DTA	Ablation of Cre-expressing PPG neurons	UNC Vectorcore	pAAV-mCherry-flex-dtA was a gift from Naoshige Uchida.
AAV2-DIO-hM_4_Di:mCherry	Inhibition of Cre-expressing PPG neurons	VVF, ZNZ, Zurich	pAAV-hSyn-DIO-hM4D(Gi)-mCherry was a gift from Bryan Roth [67]
AAV1/2-FLEX-Perceval	Control for viral transduction	Made in house.	pAAV-FLEX-empty was a gift from Bill Wisden [68] pShuttleCMV-Perceval was a gift from Guy Rutter [69]
AAV8-DIO-EGFP	Control for viral transduction	VVF, ZNZ, Zurich	pAAV-hSyn-DIO-EGFP was a gift from Bryan Roth
Chicken anti-GFP; Alexa488 goat anti- chicken; 1:1000	GCaMP3, EGFP, YFP, Perceval	Abcam AB13970; Invitrogen #A-11039	[42]
Rabbit anti-dsRed; Cy3 sheep anti-rabbit; 1:1000	mCherry, tdRFP	Takara Bio #632496; Sigma #C2306	[38]
Rabbit anti-cFOS 1:500; Cy3 sheep anti-rabbit 1:1000	cFOS	Merck #ABE457; Sigma #C2306	

#### 2.2.2 ECG recordings in anaesthetized mice

Needle electrodes were inserted subcutaneously in a lead II configuration (bilaterally at the anterior axillary lines with the ground electrode inserted in the left or right lower limb) to record surface electrocardiogram (ECG). The signal was recorded through a high impedance headstage (NL 100, Neurolog; Digitimer Ltd, UK), sampled at 2 kHz, amplified × 50–100, and filtered to a bandwidth between 5 and 100 Hz with 50 Hz notch filtering. ECG traces were recorded in Spike2 software (CED) and HR was derived from the R–R interval of the ECG. The frequency of the R wave was averaged over 5 s and was plotted as a graph in beats per minute.

HR data were exported from Spike2 into Microsoft Excel. Baseline HR was defined as the mean HR over a 10-min dataset prior to the start of the experiment. At each timepoint mean HR was extracted by averaging the HR at timepoint ± 30 s to remove the effect of any possible interference in the trace. Drug-induced changes in the mean level of activity were normalized with respect to the baseline level (ΔHR).

Drugs were delivered via i.v. or i.p. injections or directly onto the exposed thoracic spinal cord. For application directly onto the spinal cord, the middle thoracic vertebrae were exposed and the T8 lateral processes were bilaterally clamped and fixed to a stereotaxic spinal unit. The ligamentum flavum was removed between T7 and T8 and the dura mater was incised with a 21 G needle. Cerebrospinal fluid outflow was used as an indicator of access to the subarachnoid space and correct exposure of the dorsal surface of the thoracic spinal cord.

### 2.3 Awake preparations

#### 2.3.1 Tail-cuff blood pressure measurements

ABP and HR of ten 7- to 10-week-old male Glu-YFP mice were measured using the CODA high throughput volume-pressure recording (VPR) tail-cuff system (Kent Scientific). Mice were placed in a perforated plexiglass tube, fixed tightly in place, and placed on a 32 °C heat pad. An occlusion cuff and a VPR cuff were placed over the tail and 25 measurement cycles were taken. Average HR and ABP were calculated from a minimum of 10 successful measurements.

#### 2.3.2 Biotelemetry probe implantation and stereotaxic injections

Biotelemetry was used to monitor ABP and HR in conscious, freely-moving animals. Male 7-10-week-old Glu-Cre mice were anaesthetized with 1.5–2.5% isoflurane and the left common carotid artery was exposed. A gel-filled catheter connected to a pressure transmitter (TA11-PA-C10, Data Science International) was inserted and secured with sutures. The transmitter was placed subcutaneously on the abdominal wall and the incision was closed using 6–0 absorbable suture. The animals received buprenorphine (0.05 mg/kg per day, subcutaneous) and were allowed to recover for at least seven days before ABP recordings started. All mice were kept in individual cages after implantation. Two to four weeks after telemetry implantation, mice were subjected to stereotaxic injection of AAVs, as described under 2.2.1.

#### 2.3.3 Biotelemetry recordings

ABP and activity were recorded continuously for 24 h in conscious freely-moving mice. Data were acquired at a sampling rate of 500–1000 Hz and these raw data were pre-processed using a proprietary algorithm (embedded in Dataquest ART software; Data Science International) to calculate HR and mean arterial blood pressure (MAP) in 10-s time bins. These data were subsequently exported to Microsoft Excel and The R Project for Statistical Computing, R 3.3.1 (R Core Team 2016) for further analysis.

Continuous traces displaying HR or MAP from a single representative mouse over extended periods of time were smoothed using a running average to be able to recognise trends. Each data point (every 10 s) is the average of the preceding period of data points. The length of this period is given in the individual figure legend.

Plotting the distribution of HR values for individual mice (Figure S1B,C) revealed that HR values (and MAP; not shown) are not normally distributed but rather form a bimodal distribution comprising of two unimodal, non-normal distributions, which can be separated based on activity levels (Figure S1B,C). Consequently, the median, rather than the mean, for each mouse is used as a measure of resting or active HR or MAP. For population data, the mean ± SEM of these median values is calculated and displayed. One individual factor that strongly correlates with HR is activity (Figure S1C). As a result, HR and MAP were analysed separately during rest and during activity. To determine resting values for HR and MAP, Dataquest ART datasets from across the 24-h recording period were screened for periods of inactivity of at least 10 min. Since HR and MAP do not return to baseline immediately following periods of locomotor activity, the first three minutes of every 10-min period of inactivity were disregarded in order to avoid any contamination of HR and MAP from recent activity. HR and MAP values from the last 7 min of these inactive periods were combined and the median determined as a measure of resting HR for each mouse.

##### 2.3.3.1 Cardiovascular effects of Ex-4 in freely-moving mice

Four hours into the light phase, mice received an i.p. injection of either GLP-1 (100 μg/kg), Ex-4 (10 μg/kg) or saline. For studies using sympathetic blockade, mice were injected with the β1-adrenoreceptor antagonist atenolol (2 mg/kg, i.p.) 3 h and 45 min into the light phase followed 15 min later by an injection of Ex-4 or vehicle (saline). All injections were delivered in a volume of 100 μl and experiments were performed using a within-subjects counterbalanced design, with a minimum 48-h washout period between experiments. The doses of GLP-1 (100 μg/kg) and Ex-4 (10 μg/kg) were chosen based on previous studies reporting significant effects on food intake [[45], [46], [47], [48], [49]].

##### 2.3.3.2 Pharmacogenetic activation of PPG^NTS^ neurons

EGFP- or hM3Dq-expressing male Glu-cre mice were injected 7.5 h into the light phase with clozapine-N-oxide (CNO, 2 mg/kg in 5 ml/kg saline; i.p.) or with saline only in a within-subjects counterbalanced design. HR, MAP and level of activity were recorded over the following 24 h, with a minimum 48-h washout period between experiments.

##### 2.3.3.3 Cardiovascular effects of PPG^NTS^ neuron ablation

For this longitudinal study, baseline HR, MAP and level of activity were recorded and two weeks later, male Glu-Cre mice were stereotaxically injected into the NTS with either AAV8-mCherry-FLEX-DTA or AAV1/2-FLEX-Perceval as control. HR and MAP were recorded again four and six weeks after viral gene delivery. At nine weeks, DTA and control animals received i.p. injections of saline and 10 μg/kg Ex-4 as described above.

### 2.4 Histological reconstruction

At the end of the experiments, mice were transcardially perfused with 0.1 M phosphate buffer (PB) followed by 4% formaldehyde in 0.1 M PB and coronal brainstem sections (30 μm) were immunostained for the fluorescent reporters mCherry, EGFP, or Perceval as markers for successful targeting of the PPG^NTS^ neurons, as described previously [8,42]. Details of antisera used are given in Table 1. All mice were found to be expressing the expected transgene. DTA ablation of PPG neurons was confirmed by the absence of GCaMP3 immunoreactive cell bodies in the NTS, together with widespread mCherry expression, signifying viral spread. Transduction with hM3Dq and hM4Di was confirmed by mCherry expression. Selective Cre-dependent expression of all viruses in PPG^NTS^ neurons used in the present study was demonstrated previously [8] and confirmed by examination of the co-expression of virally-encoded mCherry and the transgenic label GCaMP3. From a subset of sections (n = 3 mice), this was quantified as 10.2% of Glu-Cre cells not transduced by the virus, and 13.5% of virus-transduced cells not expressing GCaMP3. This confirmed >85% co-expression.

### 2.5 cFos expression in PPG neurons

Twelve 16-week-old Glu-YFP mice of either sex received an i.p. injection of saline or Ex-4 (10 μg/kg) 4 h into the light phase and 90 min later were transcardially perfused with 0.1 M PB followed by 4% formaldehyde in 0.1 M PB. Brainstem tissue was processed and stained for cFOS and YFP, as described before [8]. Details of antisera and dilutions are given in Table 1.

### 2.6 Statistics

Data were analysed for statistical significance as detailed in figure legends using Student's *t*-test, one-way within-subjects or two-way within-subjects/mixed-model ANOVA (with the Greenhouse-Geisser correction applied where necessary), or non-parametric equivalents as appropriate. Significant one-way ANOVA tests were followed by pairwise comparisons with Tukey's or Dunnet's correction for multiple comparisons. For two-way ANOVA, simple main effects were reported, or significant treatment × time interactions were followed by analysis of treatment effects at each time point with Sidak's correction for multiple comparisons.

## 3 Results

### 3.1 Systemic exendin-4 increases heart rate in freely behaving and anaesthetized mice

HR, MAP, and activity level were measured in awake, freely behaving male mice by using implantable biotelemetry probes (Figure S1A). Conscious mice had a resting HR of 505 ± 12 bpm, and a resting MAP of 103 ± 1 mmHg.

**Figure 1:**
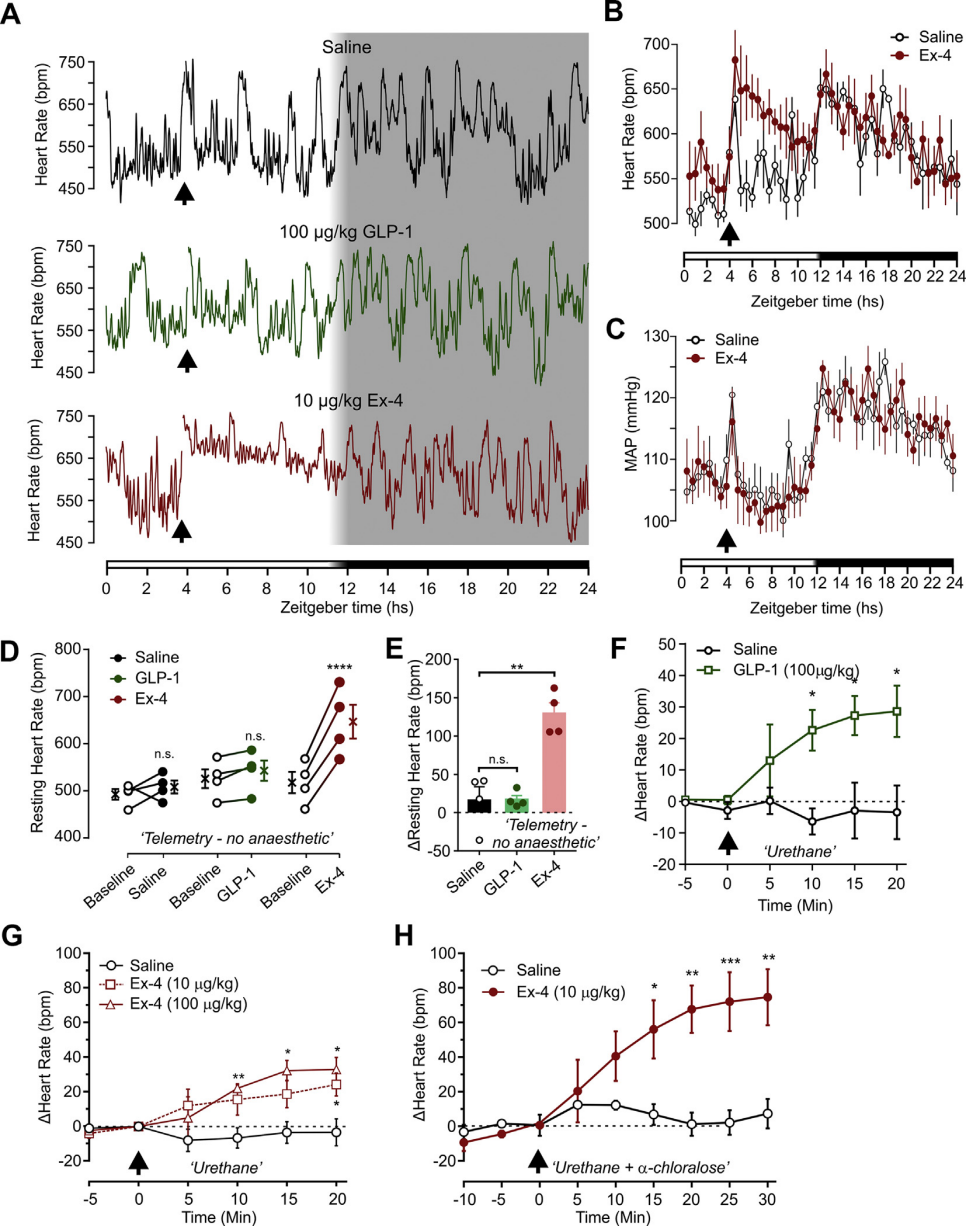
**Systemic activation of GLP-1Rs induces tachycardia without affecting ABP.** A) Representative HR recording from a conscious, freely-moving mouse injected i.p. with either saline (black, top), 100 μg/kg GLP-1(7–36) (GLP-1, green, middle), or 10 μg/kg Ex-4 (red, bottom) recorded over 24 h as indicated by zeitgeber time at the bottom. Arrows indicate times of injection and the bar at the bottom indicates light (white bar) and dark (black bar) phases with a brief period of half-light indicated by a white to black gradient. Traces are running averages over 200 s. B) HR and C) MAP of mice (n = 4) injected i.p. with saline (black) and 10 μg/kg Ex-4 (red) 4 h into light phase (arrow). Shown here are mean ± SEM of four mice taken every 30 mins over 24 h as indicated by Zeitgeber time. Bars at the bottom indicate light (white bar) and dark (black bar) phases with a brief period of half-light indicated by a white to black gradient. D) Mice implanted with biotelemetry probes were injected i.p. with saline (black), 100 μg/kg GLP-1 (green), and 10 μg/kg Ex-4 (red) 4 h into light phase. Resting HRs of individual mice were determined before (2–3 h) and after (5–6 h) i.p. injections. Paired data from individual mice are plotted as well as mean ± SEM values (n = 4). Drug x time: *F*_(1, 3)_ = 22.35, p = 0.018. ∗∗∗∗p < 0.0001, n.s.: not significant, Sidak's matched pairs multiple comparisons test. E) Change in resting HR from baseline determined from D) following injections of saline (black), GLP-1 (green), and Ex-4 (red). Data from individual mice are shown as well as mean ± SEM for each treatment (n = 4). ∗∗p < 0.01, n.s.: not significant, Dunnett's multiple comparisons test. F) Change in HR from baseline in urethane-anaesthetized mice injected i.v. at timepoint 0 (arrow) with either saline (n = 5) or GLP-1 (100 μg/kg, n = 5), Two-way mixed-model ANOVA: Drug x time *F*_(5, 40)_ = 4.824, p = 0.0015, followed by post-hoc Dunnett's test (∗p < 0.05). G) Change in HR from baseline in urethane-anaesthetized mice injected i.p. with either saline (n = 3) Ex-4 (10 μg/kg, n = 3) or Ex-4 (100 μg/kg, n = 3) at timepoint 0 (arrow). Two-way mixed model ANOVA: Drug x time *F*_(8, 28)_ = 2.53, p = 0.0329, followed by post-hoc Dunnett's test (∗p < 0.05, ∗∗p < 0.01 vs saline). H) Change in HR from baseline in urethane/α-chloralose-anaesthetized mice injected i.p. with either saline (n = 3) or Ex-4 (10 μg/kg, n = 3) at timepoint 0 (arrow). Two-way mixed-model ANOVA: Drug x time *F*_(6, 36)_ = 7.459, p < 0.0001, followed by Sidak's multiple comparisons test (∗p < 0.05, ∗∗p < 0.01, ∗∗∗p < 0.001).

Injection of 100 μl saline i.p. led to a rapid increase in HR (Figure 1A,B) and MAP (Figure 1C) as expected due to handling of the mouse and noxious stimulation induced by the injection. Both HR and MAP returned to baseline over 30 min and continued to fluctuate as normal with more frequent returns to high values during the dark phase when the mice are more active (Figure 1A–C, Figure S1A,C). Similarly, i.p. injection of 100 μg/kg GLP-1 caused a transient increase in HR, which returned to baseline within 30 mins (Figure 1A). In contrast, i.p. injection of the long-acting GLP-1 analogue Ex-4 (10 μg/kg) led to a sustained increase in HR over the remainder of the light phase (Figure 1A,B), whereas MAP was unaffected by systemic GLP-1R activation (Figure 1C).

Ex-4 increased the minimum level of HR, whilst peak rates remained unchanged, suggesting that the effect was mainly on resting HR (Figure. 1A). Consequently, resting HRs were calculated over 1 h before (Zeitgeber 2–3 h) and after (Zeitgeber 5–6 h) drug treatments. Ex-4 (10 μg/kg, i.p.) significantly increased resting HR (Figure 1D,E), corroborating previous findings that GLP-1R activation increases mean HR [24,25,50], without affecting activity levels (Figure S1D-F). There was no effect of saline or GLP-1 on resting HR during the time window analysed.

Given that Ex-4 significantly increased resting HR, and since the effects of GLP-1 were potentially masked by the cardiovascular stress response, we explored the effects of systemic GLP-1R activation under anaesthesia, when stress is not a confounding factor. Infusion of GLP-1 (100 μg/kg; i.v.) and two doses of Ex-4 (10 and 100 μg/kg; i.p.) significantly increased HR with comparable amplitudes (Figure 1F,G). Interestingly, the effect of 10 μg/kg Ex-4 was substantially smaller under anaesthesia than in conscious mice (Figure 1E,G). We reasoned that urethane anaesthesia might increase sympathetic tone [51] and thus reduce the scope for GLP-1R activation to increase HR via an increase in sympathetic outflow. This hypothesis was tested by applying Ex-4 (i.p.; 10 μg/kg) in mice anaesthetized with urethane (650 mg/kg) + α-chloralose (50 mg/kg), an anaesthetic regime which maintains a lower sympathetic tone [51]. Resting HR under these conditions was significantly lower than under urethane alone and close to the resting HRs recorded with biotelemetry in conscious mice (Figure S2A) and i.p. injection of Ex-4 (10 μg/kg) triggered HR responses that were almost three times greater than under urethane alone (Figure 1H,G, respectively).

### 3.2 Systemic GLP-1R activation increases heart rate via the sympathetic nervous system

**Figure 2:**
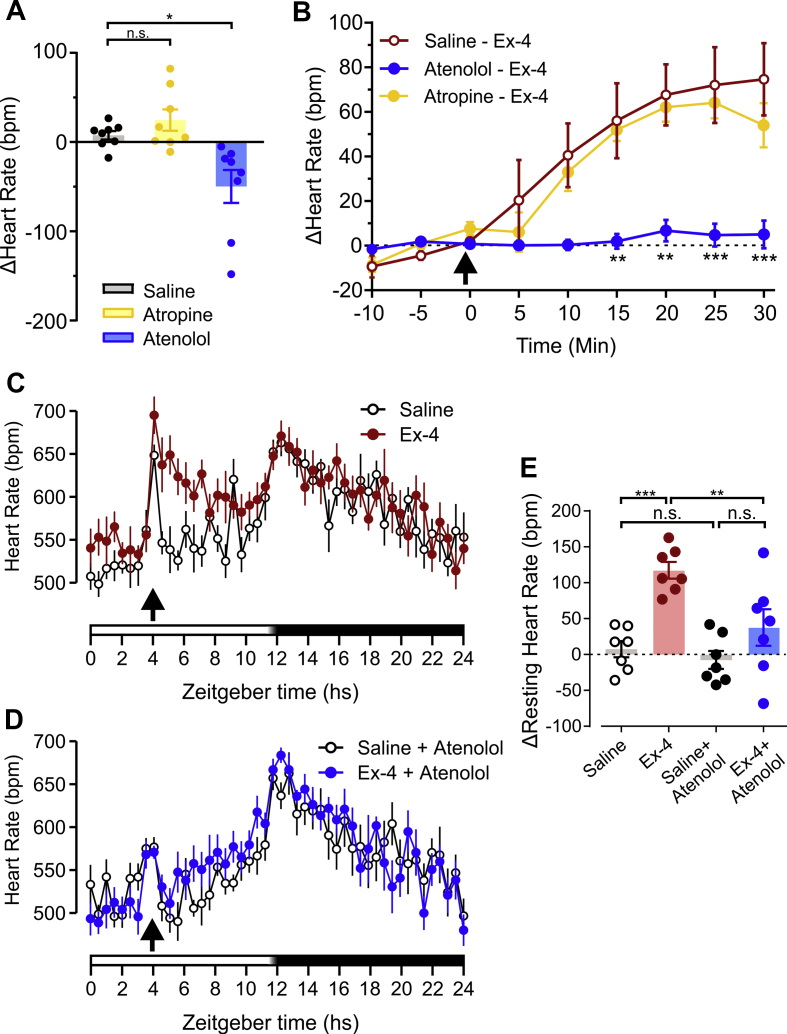
**Systemic GLP-1R activation induces tachycardia via the sympathetic nervous system in anaesthetized and freely behaving mice.** A) Change in HR in urethane/α-chloralose-anaesthetized mice 25 min after i.p. injection of saline (n = 8), atropine (2 mg/kg, n = 8) or atenolol (2 mg/kg, n = 8). One-way ANOVA *F*_(2,21)_ = 8,808, p = 0.0017, followed by post-hoc Dunnett's test (∗p < 0.05) B) Change in HR from baseline in anaesthetized mice following i.p. injection of Ex-4 (10 μg/kg; arrow) in the absence (n = 4, Sal-Ex-4) or presence (n = 4, Aten-Ex-4) of atenolol (2 mg/kg, i.p.) injected 30 mins earlier. Two-way mixed model ANOVA: drug x time *F*_(12, 54)_ = 3.562, p = 0.0006, followed by post-hoc Dunnet's test (∗∗p < 0.01, ∗∗∗p < 0.001 vs Sal-Ex4). C, D) Mice implanted with biotelemetry probes (n = 7) were injected i.p. with saline or Ex-4 (10 μg/kg, arrow) 4 h into light phase in the absence (C) or presence (D) of atenolol (2 mg/kg) injected 15 mins earlier. Shown here are mean ± SEM taken every 30 mins over 24 h as indicated by zeitgeber time on the x-axis. Arrows indicate time of injection and the bars at the bottom indicate light (white bar) and dark (black bar) phases with a period of half-light indicated by a white to black gradient. E) Change in HR (1–2 h post-injection) from baseline (1–2 h before injection) in biotelemetry probe-implanted mice in response to saline or Ex-4 (10 μg/kg) in the absence or presence of atenolol (2 mg/kg, n = 7). Pre-treatment x drug: *F*_(1, 6)_ = 13.84, p = 0.0099, ∗∗p > 0.01, ∗∗∗p < 0.001 and n.s.: not significant, Sidak's multiple comparisons test.

To determine whether the increase in HR by Ex-4 is due to increased sympathetic outflow or decreased vagal tone, anaesthetized mice were injected (i.p.) with either the β-adrenoceptor antagonist atenolol (2 mg/kg) or the muscarinic receptor antagonist atropine (2 mg/kg) [52] 30 min before Ex-4 (10 μg/kg) or saline. Atropine failed to significantly alter HR (Figure 2A), suggesting that parasympathetic activity has no contribution to chronotropic control of the heart under these experimental conditions. In contrast, atenolol decreased HR (Figure 2A), however there was no significant difference in baseline HR between Atenolol/Saline- and Atenolol/Ex-4-treated mice at the time of injections (Figure S2B). The tachycardic response to Ex-4 was abolished by systemic β-adrenoceptor blockade (Figure 2B) but not affected by pre-treatment with atropine (Figure 2B), suggesting that the Ex-4-induced HR increase is due to sympathetic activation.

We subsequently confirmed those findings in freely-behaving, awake mice. Injection of 10 μg/kg Ex-4 alone induced a sustained increase in resting HR over several hours (Figure 2C,E). Pre-treatment with 2 mg/kg atenolol reduced the effect of handling stress on resting HR consistent with a decrease in sympathetic outflow to the heart (Figure 2D). Ex-4 failed to increase resting HR in conditions of systemic β-adrenoceptor blockade (Figure 2D,E).

### 3.3 Chemogenetic activation of PPG^NTS^ neurons

**Figure 3:**
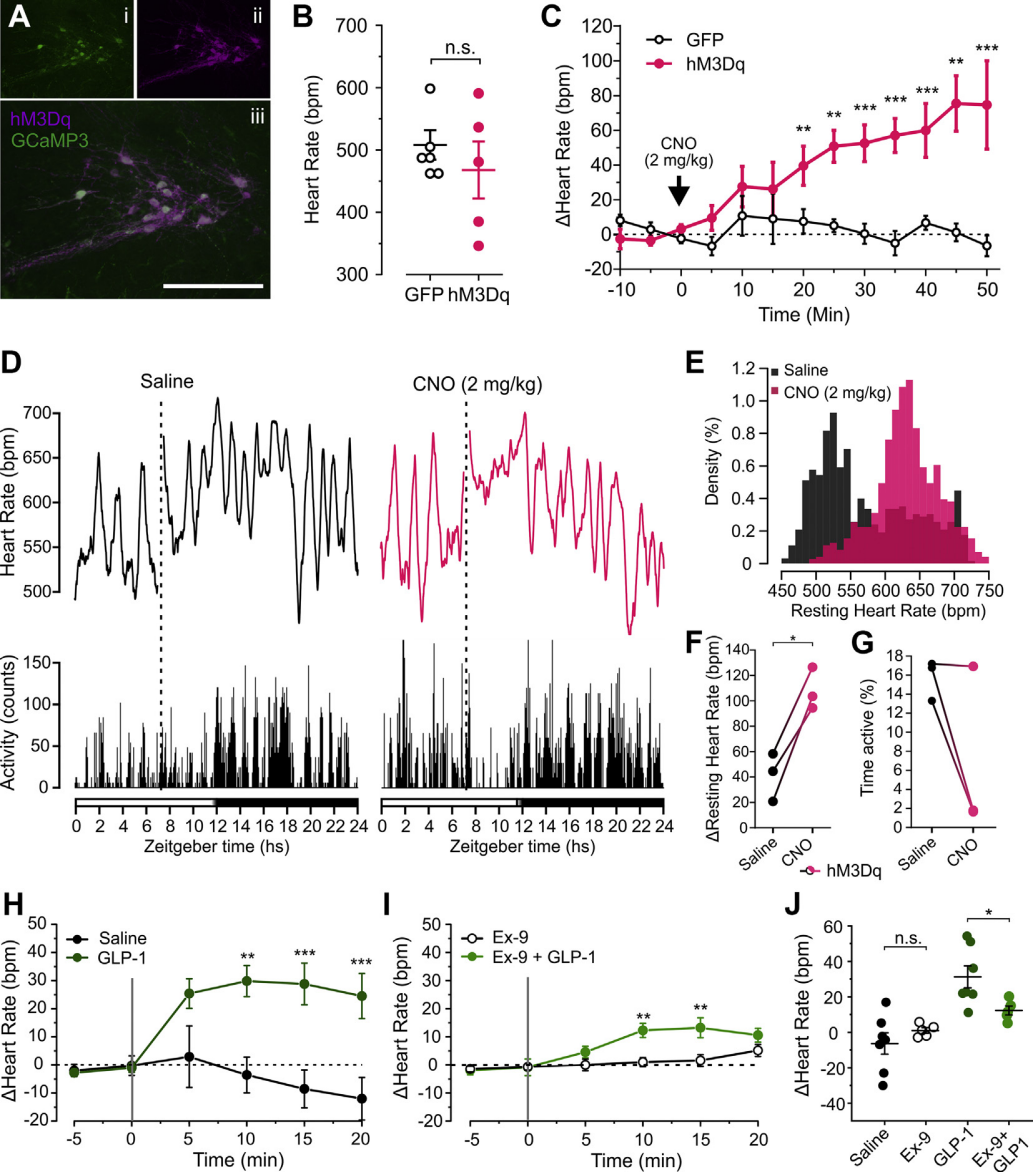
**Chemogenetic activation of PPG**^**NTS**^**neurons and direct application of GLP-1 to the spinal cord increase HR in mice.** A) Representative images of immunofluorescence double-labelling of GCaMP3 (green, indicating PPG^NTS^ neurons) and hM3Dq:mCherry (magenta) in the caudal NTS. Scale bar: 200 μm B) Baseline HR prior to injection of CNO from urethane/α-chloralose-anaesthetized mice expressing either GFP or hM3Dq in PPG^NTS^ neurons (unpaired t-test). C) Change in HR from baseline in response to CNO (2 mg/kg) injected after 10 mins baseline recording (arrow) from urethane/α-chloralose-anaesthetized mice expressing either GFP or hM3Dq in PPG^NTS^ neurons. Two-way ANOVA: Virus x time *F*_(9, 72)_ = 5.477, p < 0.0001; followed by Sidak's multiple comparisons test (∗∗p < 0.01, ∗∗∗p < 0.001). D) Representative 24 h HR (top) and locomotor activity (bottom) recording of a freely-behaving mouse expressing hM3Dq in PPG^NTS^ neurons injected i.p. with either saline (left) or 2 mg/kg CNO (right). Dotted lines indicate times of injection and the bar at the bottom indicates light (white bar) and dark (black bar) phases with a brief period of half-light indicated by a white to black gradient. Traces are running averages over 30 mins. E) Representative histogram showing densities of resting HR values following i.p. injections of either saline (black) or 2 mg/kg CNO (magenta). Resting HR values after saline and CNO, respectively, were taken from 8.5 to 10.5 h (1–2 h post-injection) to avoid contamination of resting HRs with HR values during handling stress. Total density areas of individual histograms are 100. F, G) Change in resting HR (F) and percent of time spent active (G) of hM3Dq-expressing mice (n = 3) in response to saline (black circles) and 2 mg/kg CNO (magenta circles). ∗p < 0.05, paired t-test. H) Change in HR (mean ± SEM) from baseline of urethane/α-chloralose-anesthetized mice in response to application of saline (n = 7) or GLP-1 (0.4 μg, n = 7) directly onto the thoracic spinal cord. Two-way ANOVA: Drug x time *F*_(5, 55)_ = 7.18, p < 0.0001, followed by Sidak's post-hoc multiple comparisons test (∗∗p < 0.01, ∗∗∗p < 0.001 vs saline). I) Change in HR (mean ± SEM) from baseline of urethane/α-chloralose-anaesthetized mice in response to application of Ex-9 (18.75 μg, n = 5), or by GLP-1 (0.4 μg, n = 5) 25 min after Ex-9, directly onto the thoracic spinal cord. Two-way ANOVA: Drug x time *F*_(5, 40)_ = 3.971, p = 0.0051, followed by Sidak's post-hoc multiple comparisons test (∗∗p < 0.01 vs Ex-9). J) Change in HR 10 min after application of each drug. Ex-9 applied to the thoracic cord did not significantly change HR, but significantly reduced the effect of subsequently applied GLP-1. Two-way mixed model ANOVA: GLP-1 x Ex-9 *F*_(1,10)_ = 5.525, p = 0.0406, followed by Sidak's post-hoc multiple comparisons test (∗p < 0.05).

Within the brain, PPG^NTS^ neurons are the main source of endogenous GLP-1 [8]. To study their role in the modulation of HR, male Glu-Cre/GCaMP3 mice were injected with AAV8-DIO-hM3Dq:mCherry into the NTS (Figure 3A) and once hM3Dq was expressed, HR was monitored under urethane/α-chloralose anaesthesia. Baseline HR did not differ between mice transduced to express GFP and those transduced to express hM3Dq (Figure 3B). Activation of PPG^NTS^ neurons with CNO (2 mg/kg; i.p.) led to a significant increase in HR in hM3Dq-expressing mice but not GFP-expressing mice (Figure 3C).

Next, we confirmed that PPG^NTS^ neuron activation is sufficient to increase HR in freely behaving mice. Male Glu-Cre mice were injected with AAV8-DIO-hM3Dq:mCherry, and biotelemetry blood pressure probes were implanted. As a positive control for successful transduction, food intake was measured following CNO or saline as previously described [8]. Injection of CNO significantly suppressed feeding in the first 2 h after dark onset as compared to injection of saline only (Figure S2D).

Injection of CNO led to a sustained increase in HR compared to injection of saline only (Figure 3D) and resulted in a shift in the distribution of resting HR values towards higher HR values (Figure 3E). This was reflected in the resting HR of hM3Dq-epxressing mice, which increased significantly following injection of CNO compared to saline only (Figure 3F). Although a reduction in locomotor activity was observed in two hM3Dq-expressing mice following 2 mg/kg CNO as compared to saline, this decrease did not occur in the third hM3Dq-expressing mouse (Figure 3G).

### 3.4 Ex-4 elicits tachycardia when applied directly to the spinal cord

Given that a subset of PPG neurons projects to the spinal cord and forms close appositions with sympathetic preganglionic neurons [37], and GLP-1R expression has been reported by *in situ* hybridisation within the spinal cord [40], we tested whether GLP-1 signalling in the cord can modulate HR. GLP-1 (0.4 μg in 2.5 μl saline) was applied directly to the exposed spinal cord in anaesthetized mice, which significantly increased HR as compared to application of saline alone (Figure 3H). Local application of the GLP-1R antagonist exendin (9–39) (18.75 μg in 2.5 μl saline) did not affect HR, indicating that there is no tonic GLP-1 activity in the spinal cord, but it strongly reduced the effect of subsequent application of GLP-1 (0.4 μg) on HR (Figure 3I,J).

### 3.5 Resting HR is not affected by either acute inhibition or ablation of PPG^NTS^ neurons

**Figure 4:**
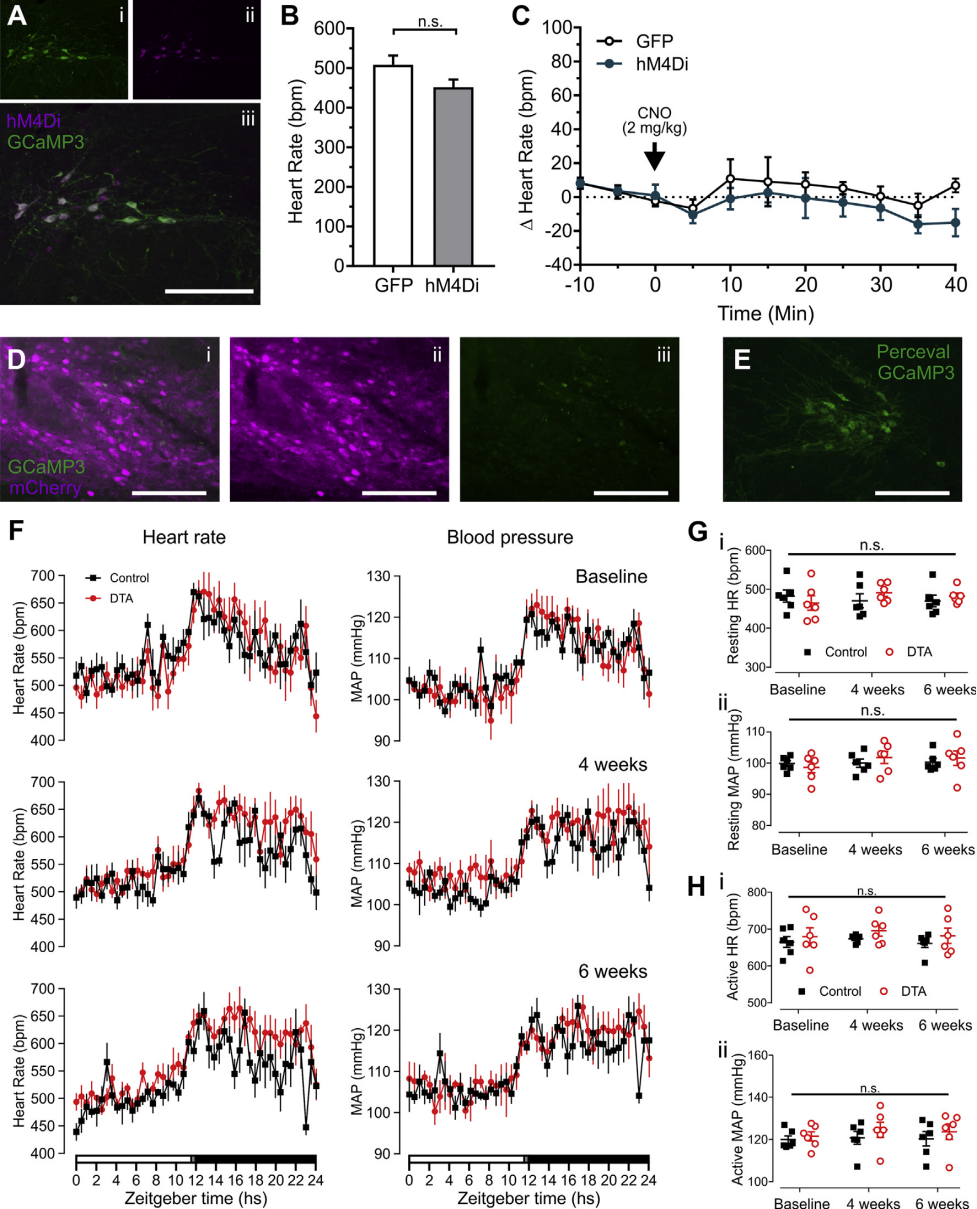
**Inactivation or ablation of PPG**^**NTS**^**neurons does not affect HR or MAP.** A) Representative images of immunofluorescence double-labelling of GCaMP3 (green, indicating PPG^NTS^ neurons) and hM4Di:mCherry (magenta) in the caudal NTS. B) HR at baseline for control (GFP) and hM4Di expressing mice prior to CNO administration. C) Change in HR from baseline of anaesthetized mice expressing either GFP (n = 5) or hM4Di (n = 3) in PPG^NTS^ neurons selectively in response to CNO (2 mg/kg) injected at 0 mins (arrow). Virus x time: *F*_(8, 48)_ = 0.196, p = 0.9902. D) representative photomicrographs of immunofluorescence double-labelling of GCaMP3 (green, indicating PPG neurons) and mCherry (magenta) expressed cre-independently from AAV8-mCherry-FLEX-DTA in the caudal NTS. Note the presence of green fluorescent debris, much smaller than cell bodies, only. E) Representative image of immunofluorescence double-labelling of GCaMP3 and Perceval (both green) in the caudal NTS, indicating presence of PPG^NTS^ neurons in mice injected with control virus for ablation experiments. All scale bars: 200 μm. F) HR (panels on left) and MAP (panels on right) were recorded before DTA ablation of PPG^NTS^ neurons (Baseline, top panel) and at four (middle panel) and six weeks (bottom panel) after surgery. Traces display mean ± SEM HR and ABP every 30 mins for control (black squares; n = 6) and DTA mice (red circles; n = 6). Light (white bar), half light (gradient bar) and dark phase (black bar) are indicated at the bottom along with Zeitgeber time. G, H) Resting (G) and active (H) HR and MAP of control and PPG^NTS^-DTA (DTA) mice at baseline, four weeks, and six weeks after surgery. n.s.: no significant interaction or main effects according to two-way mixed model ANOVA: Resting HR: Virus x time: *F*_(2, 20)_ = 2.178, p = 0.14; main effects of virus (p = 0.78) and time (p = 0.74). Resting MAP: Virus x time: *F*_(2, 20)_ = 1.522, p = 0.2426; main effects of virus (p = 0.77) and time (p = 0.14). Active HR: Virus x time: *F*_(2, 20)_ = 0.0835, p = 0.9202; main effects of virus (p = 0.36) and time (p = 0.37). Active MAP: Virus x time: *F*_(2, 20)_ = 0.1653, p = 0.85; main effect of virus (p = 0.43) and time (p = 0.65).

Although chemogenetic activation of PPG^NTS^ neurons induced tachycardia in mice, this does not prove these neurons play a physiological role in cardiovascular control. We therefore acutely inhibited PPG^NTS^ neurons in hM4Di-expressing mice anaesthetized with urethane and α-chloralose. Male Glu-Cre/GCaMP3 mice stereotaxically injected with AAV2-DIO-hM4Di:mCherry or with AAV8-DIO-EGFP (Figure 4A) had similar HRs prior to injection of CNO (2 mg/kg i.p.; Figure 4B), and CNO had no effect on HR (Figure 4C).

We next tested whether ablation of these cells has a significant effect on cardiovascular variables in a longitudinal study. Male Glu-Cre mice implanted with biotelemetry probes were stereotaxically injected with AAV8-mCherry-FLEX-DTA (Fig 4Di-iii, S2F; PPG^NTS^-DTA mice) to selectively ablate PPG^NTS^ neurons as previously described [8] or AAV1/2-FLEX-Perceval as control (Figure 4E, Figure S2G). We have demonstrated that ablation of PPG^NTS^ neurons in this manner results in an 80% reduction in GLP-1 within the spinal cord [8].

Prior to PPG^NTS^ neuron ablation, there was no difference in HR and MAP recorded over 24 h between the two cohorts (Figure 4F). Similarly, when tested four and six weeks after PPG^NTS^ neuron ablation, no significant difference was seen between control and PPG^NTS^-DTA mice at either timepoint (Figure 4F).

Because GLP-1R activation by Ex-4 and PPG^NTS^ chemogenetic activation were found to increase HR at times of rest, we conducted a further analysis to determine whether the ablation of PPG^NTS^ neurons affected resting HR in these mice (Figure 4G). Prior to ablation, there was no difference in resting HR between control and PPG^NTS^-DTA mice (Figure 4G). Moreover, there was little change in resting HR and MAP over time and no difference was found between control and PPG^NTS^-DTA mice at any timepoint (Figure 4G).

To investigate whether ablation of PPG^NTS^ neurons affects HR and MAP whilst ambulatory, biotelemetry data were extracted specifically during periods of activity. As expected, HR during activity was higher than resting HR (Figure 4Hi ‘control baseline’ and Figure 4Gi ‘control baseline’, respectively p < 0.0001, Student's paired *t*-test). HR during activity remained similar from baseline to six weeks and there was no difference between control and PPG^NTS^-DTA mice six weeks after surgery (Figure 4Hi). Similarly, there was no effect of ablation of PPG^NTS^ neurons on MAP during activity (Figure 4Hii).

**Figure 5:**
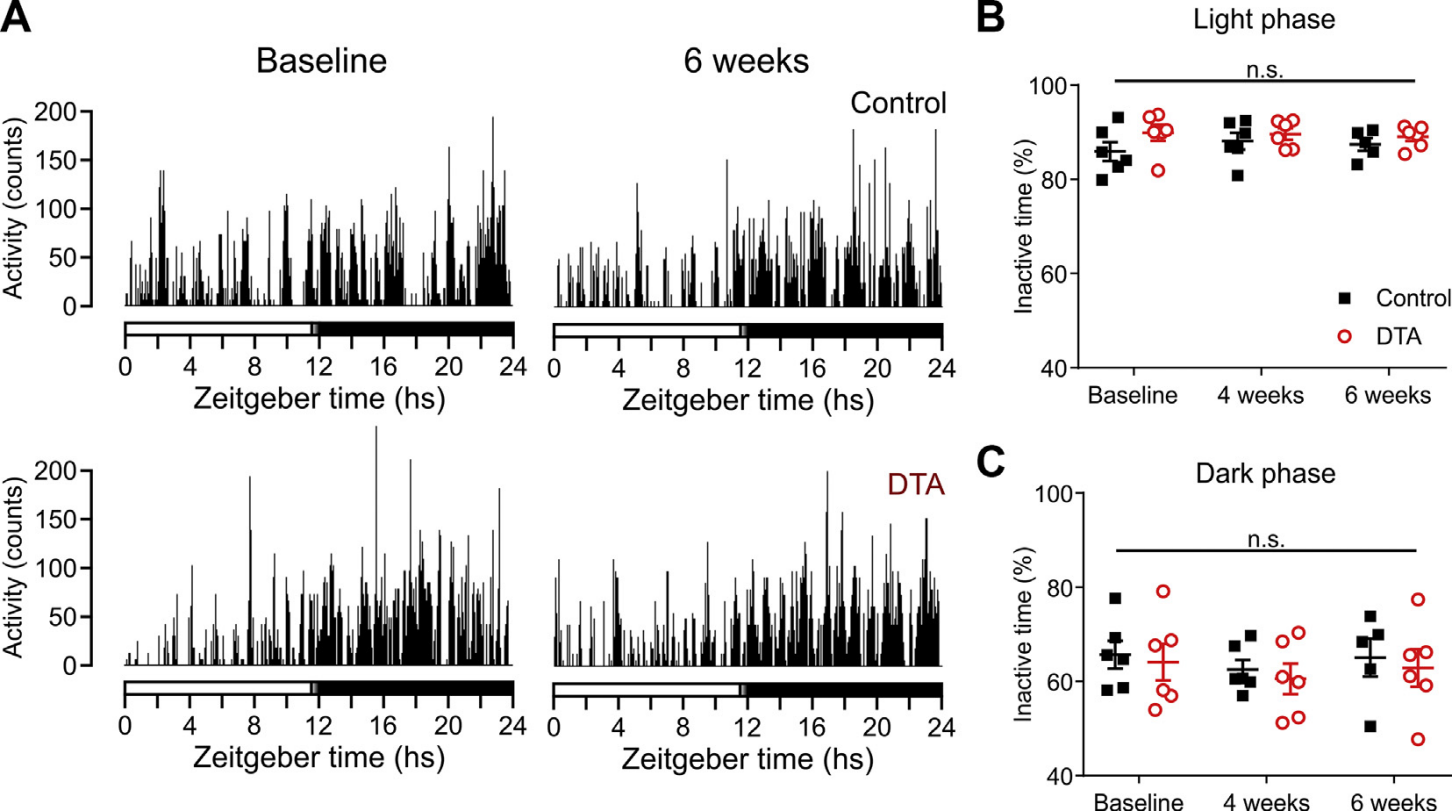
**Ablation of PPG**^**NTS**^**neurons does not affect locomotor activity in response to increased activity.** A) Activity plots from representative control (top panels) and PPG^NTS^-DTA (DTA, bottom panels) mice prior to viral injection (Baseline, left) and six weeks post-injection (6 weeks, right). B, C) Percent of time spent inactive of control (black squares) or PPG^NTS^-DTA mice (DTA, red circles) during the light (B) and dark phase (C). Virus x time (light phase): *F*_(2, 20)_ = 0.9748, p = 0.39; main effect of virus (p = 0.25) and time (p = 0.71). Virus x time (dark phase): *F*_(2, 20)_ = 0.006161, p = 0.9939); main effect of virus (p = 0.65) and time (p = 0.44).

We next assessed the effects of PPG^NTS^ neuron ablation on locomotor activity. Figure 5A shows plots of activity over 24 h from representative control and PPG^NTS^-DTA mice before and six weeks after surgery. Ablation of PPG^NTS^ neurons did not affect activity levels and six weeks post-surgery, there was no difference in the time that control and PPG^NTS^-DTA mice spent inactive during light phase (Figure 5B) or dark phase (Figure 5C), suggesting that the loss of PPG^NTS^ neurons does not affect locomotor activity.

Taken together, these data suggest that PPG^NTS^ neurons are not necessary for the modulation of HR or ABP under resting conditions. They also do not influence locomotor activity levels.

### 3.6 Ex-4-induced tachycardia is independent of PPG^NTS^ neuron activity

To investigate whether the systemic injection of Ex-4 requires PPG neurons for its full effect on HR, we injected freely-behaving control and PPG^NTS^-DTA mice with 10 μg/kg Ex-4 i.p. nine weeks post-surgery.

**Figure 6:**
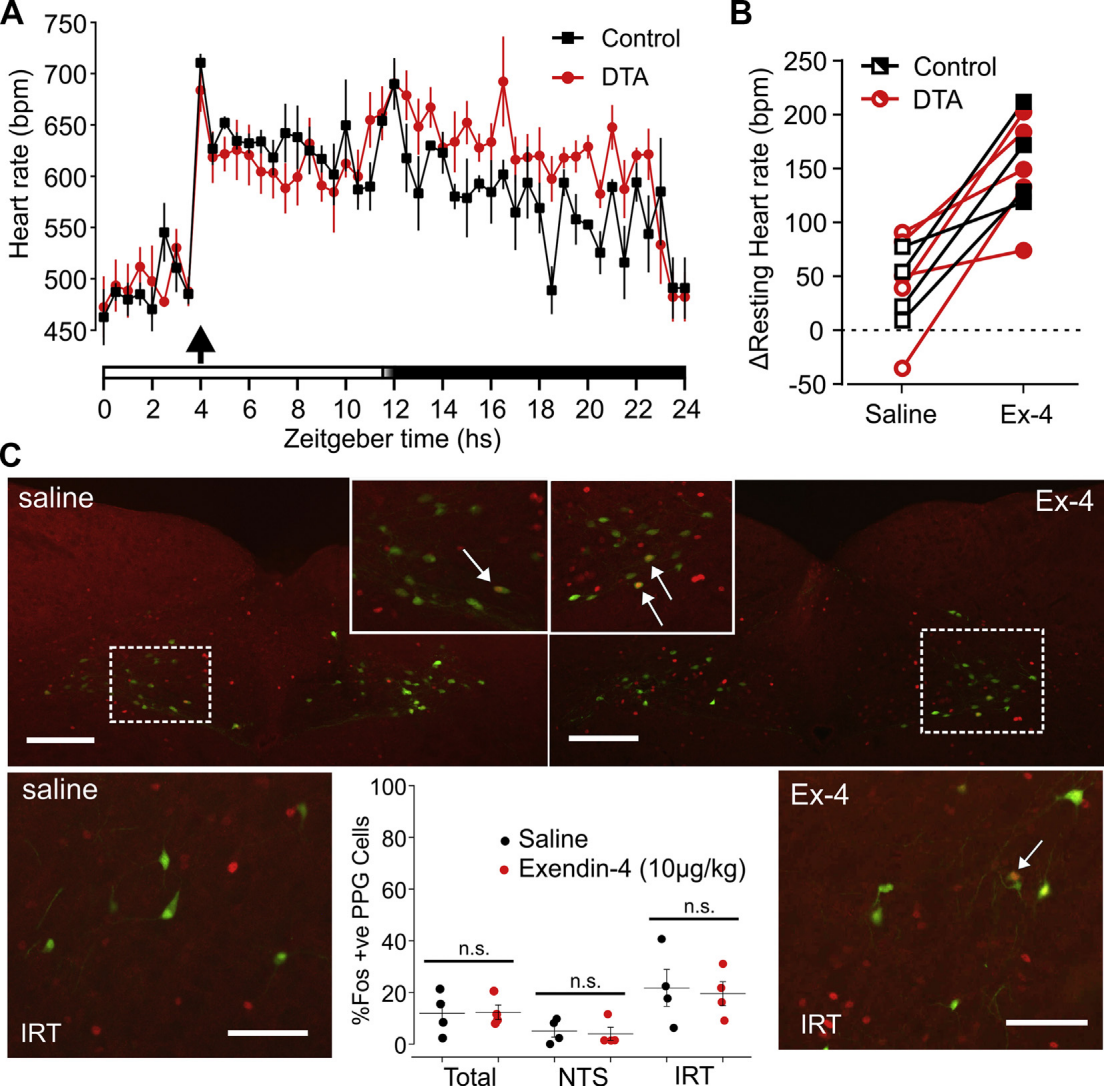
**PPG**^**NTS**^**neurons are not necessary for the tachycardic response to systemic GLP-1R activation.** A) HR of control (black squares, n = 4) and PPG^NTS^-DTA mice (DTA, red circles, n = 5) injected i.p. with 10 μg/kg Ex-4 (red) 4 h into light phase (arrow). Shown here are mean ± SEM every 30 mins over 24 h as indicated by zeitgeber time on the x-axis. The bar at the bottom indicate light (white bar) and dark (black bar) phases with a brief period of half-light indicated by a white to black gradient. B) Change in resting HR of control (black squares) and PPG^NTS^-DTA mice (red circles) in response to saline (open symbols) and 10 μg/kg Ex-4 (filled symbols). Drug x virus: *F*_(1, 7)_ = 0.3287, p = 0.5844); no significant main effect of transgene expression (p = 0.7610), but a significant main effect of treatment (p = 0.0008). C) Representative images and quantification of immunofluorescence labelling of cFos (red) in the caudal NTS (top panels) and IRT (bottom panels) following i.p. injection of saline or Ex-4 (10 μg/kg). Green indicates native YFP immunofluorescence in PPG neurons. Scale bars: 100 μm. Quantification plots show mean ± s.e.m of % cFos-IR positive PPG neurons in NTS and intermediate reticular nucleus (IRT) for 4 mice under each condition. Unpaired t-tests revealed no significant differences between saline- and Ex-4-treated mice.

As seen previously, i.p. injection with 10 μg/kg Ex-4 led to an increase in HR (Figure 6A). The response was similar in control and PPG^NTS^ ablated animals (Figure 6A), and there was no difference in the change in resting HR in response to both saline and 10 μg/kg Ex-4 between control and PPG^NTS^-DTA mice (Figure 6B, Figure S2E). In support of these findings, i.p. injection of Ex-4 (10 μg/kg) failed to elicit cFos expression in PPG neurons (Figure 6C) whilst increasing cFos in the area postrema (Figure S2H,I), suggesting PPG neurons are not necessary for effects of systemic Ex-4 on HR in mice.

## 4 Discussion

In this study, we demonstrate for the first time that the activation of PPG^NTS^ neurons induces robust increases in HR. We also confirm previous reports that systemic administration of GLP-1RAs increases HR via stimulation of sympathoexcitatory mechanisms in mice [1]. We demonstrate that GLP-1R activation in the spinal cord is sufficient to elicit tachycardic responses and we also found that PPG^NTS^ neurons are capable of increasing resting HR but not ABP. Interestingly, we failed to observe a tonic drive of PPG^NTS^ neurons on HR in either anaesthetized or freely behaving mice. Our findings provide evidence that PPG^NTS^ neurons have the capacity to affect central cardiovascular control and sympathetic activity but suggest that they do not provide a tonic input to cardiac chronotropic function.

This study was performed primarily on mixed sex cohorts, although the telemetry cohorts were made up of male mice only. The study did not reveal any sex differences in any parameters tested but was also not specifically designed nor powered for that purpose.

### 4.1 Mechanisms underlying GLP-1R-mediated tachycardia

Although the tachycardic effects of GLP-1R stimulation have proven robust and reproducible, particularly in rodents, the underlying neurocircuits remain unclear. GLP-1R-mediated cardiovascular effects may involve a combination of peripheral and central pathways involving GLP-1Rs both in the brain and on the heart [1,3,22,50,53]. Furthermore, both the parasympathetic [2,3,5,25] and sympathetic nervous system [1,13,24,29] have been implicated in the tachycardic response to GLP-1R stimulation.

The most commonly proposed scenario for GLP-1R-mediated tachycardia involves both peripheral and central mechanisms [1,3,24]. GLP-1Rs are found in subsets of cardiac myocytes, cardiac blood vessels, as well as the sinoatrial node, of mice and humans [39,54,55], and mice lacking cardiac GLP-1Rs have reduced HR responses to the GLP-1R agonist, liraglutide [1]. However, no direct effect of GLP-1R agonism on isolated hearts has been found [1,50], leaving the role of cardiac GLP-1R-mediated tachycardia elusive.

Findings presented here support a role for the sympathetic nervous system in mediating Ex-4-induced tachycardia. I.p. Ex-4 failed to increase HR in the absence of sympathetic input to the heart in both anaesthetized and freely-behaving mice. Moreover, the tachycardic response to i.p. Ex-4 persists in the presence of the muscarinic acetylcholine receptor antagonist atropine, demonstrating that parasympathetic input is not necessary for cardiovascular effects of systemic GLP-1R stimulation. These findings correspond with earlier reports that GLP-1R stimulation activates sympathetic preganglionic neurons [24,29] and that propranolol, a non-selective β-blocker, abolishes the tachycardic response to liraglutide in mice [1].

### 4.2 Peripheral and central contributions to GLP-1R-mediated tachycardia

Cardiac sympathetic nervous activity is initiated within the central nervous system, involving pre-sympathetic neurons in the hypothalamus and brainstem, which in turn innervate sympathetic preganglionic neurons in the thoracic spinal cord. These project to the postganglionic neurons located in the ganglia of the paravertebral chain, which innervate the sinoatrial node as well as the ventricles of the heart. GLP-1Rs are potentially present at all levels. Consequently, Ex-4-induced tachycardia could arise from all these levels, whilst PPG neurons only innervate pre-sympathetic areas of hypothalamus and brainstem as well as preganglionic sympathetic neurons in the IML and CAA of the spinal cord [36,37]. Here we demonstrate that direct application of Ex-4 to the thoracic spinal cord elicits tachycardia, supporting the notion that activation of spinal GLP-1R is sufficient to drive increases in HR [37,40].

Whilst Ex-4 increased HR in both anaesthetized and conscious mice, GLP-1 only produced obvious tachycardia in anaesthetized mice. This is most likely because conscious mice show a strong stress response to handling and i.p. injection, and HR only returns to resting levels more than 30 min after the injection. Presumably, GLP-1 is inactivated by that time so that no lasting response is recorded [4,24,56,57]. In contrast, in anaesthetized mice Ex-4 and GLP-1 produced responses of similar magnitude, suggesting that both substances reach the relevant receptors with similar efficiency.

### 4.3 GLP-1R effects on blood pressure

While we found clear effects of GLP-1R stimulation on HR, freely behaving mice showed no change in ABP in response to 10 μg/kg Ex-4. In accordance, one study found no effect of liraglutide on systolic and diastolic blood pressure in mice, although the same study reported antihypertensive effects of liraglutide in mice with pharmacologically elevated ABP [54]. Some studies have reported robust hypertensive effects of GLP-1 analogues in rats [24,25], whereas others found little evidence for an effect on ABP [27], suggesting that there could be relevant species differences or that differences in experimental conditions affect ABP responses to GLP-1R stimulation.

### 4.4 PPG^NTS^ neurons have the ability to induce tachycardia

We demonstrate here that chemogenetic activation of PPG^NTS^ neurons increases HR. The activation of PPG neurons is expected to lead to the release of GLP-1 and glutamate [58,59] in CNS areas involved in cardiovascular control, such as the PVN, arcuate nucleus, RVLM, CAA, and IML [38,40,60]. The dense innervation of the spinal cord IML/CAA by PPG neurons [37] and the robust effects of direct application of Ex-4 to the thoracic spinal cord reported here strongly implicate PPG^NTS→IML/CAA^ projections in PPG-mediated tachycardia. Selective viral targeting of these projections in future studies are thus warranted to interrogate their specific role in the modulation of cardiovascular function by GLP-1.

Interestingly, we found that the tachycardic effects of both Ex-4 and PPG^NTS^ neuron activation were mainly on resting HR. This may reflect a ceiling effect, whereby GLP-1R-mediated activation of the sympathetic nervous system has no additional effect on HR at times of increased ambulatory activity and/or stress. In support of this, under urethane anaesthesia, which is known to increase sympathetic outflow, Ex-4-induced tachycardia was attenuated. These data also suggest that common mechanisms underlie the cardiovascular effects of PPG^NTS^ activation and GLP-1R stimulation. Importantly, we cannot rule out that the relatively large dose of Ex-4 used in this study (10 μg/kg) may lead to higher levels of GLP-1R engagement than chemogenetic activation of PPG^NTS^ neurons, and additionally, that Ex-4 would potentially reach GLP-1Rs on cells that are not innervated by PPG neurons, such as postganglionic sympathetic neurons or cardiac myocytes, and thus replicate the effects of gut-derived GLP-1.

### 4.5 Endogenous, central GLP-1 in cardiovascular control

Whilst exogenous GLP-1 and its analogues are clearly capable of eliciting a tachycardic response, it is less clear whether endogenous GLP-1 released from either the brain or the gut plays a role in day-to-day HR regulation. This question has been addressed in two ways. First, Barragan et al. infused the GLP-1R antagonist, Ex-9 either i.v. or intracerebroventricular and found no effect on HR or ABP [3,4]. As an alternative approach, we inhibited or ablated the PPG^NTS^ neurons and thereby the native source of GLP-1 within the CNS [8]. In support of the results obtained with Ex-9, the current study revealed no effect of the loss of PPG^NTS^ neuron activity on HR or ABP under typical physiological conditions. Our findings corroborate previous reports that HR is unaffected by both genetic disruption of GLP-1R [53] and lack of hypothalamic GLP-1R [13].

### 4.6 Relationship between exogenous GLP-1R agonists and PPG neurons

Here we have shown that both the activation of PPG^NTS^ neurons and the systemic application of Ex-4 increase HR via stimulation of the sympathetic nervous system. We have also shown that peripherally-administered Ex-4 does not activate PPG neurons. This suggests that either PPG^NTS^ neurons and Ex-4 both have access to the same neuronal GLP-1R population(s) or that they activate sympathetic inputs to the heart via different pathways. The action of PPG^NTS^ neurons is limited to receptors within areas of the CNS, which receive input from PPG neurons [[35], [36], [37], [38]]. On the other hand, systemic Ex-4 may reach GLP-1R in both the periphery and in the CNS, although it is becoming increasingly evident that GLP-1RAs and antagonists may not readily cross the blood brain barrier to access all CNS GLP-1R populations [[61], [62], [63]]. In fact, assuming that access to the CNS is similar for peripherally administered Ex-4 as it is for fluorescently labelled liraglutide [62,63], we would expect that Ex-4 acts in either PVN, AP/NTS or on spinal preganglionic sympathetic neurons to affect HR. This is supported by a previous finding that peripheral Ex-4 leads to the robust activation of tyrosine hydroxylase-containing cells within the AP [29].

### 4.7 GLP-1 and stress

Inhibition of PPG^NTS^ neurons did not reduce HR in anaesthetized mice, indicating that there is no tonic effect of PPG^NTS^ neurons on sympathetic outflow to the heart under these conditions. We have recently shown that PPG^NTS^ neurons are not involved in the day-to-day regulation of food intake, but are recruited to terminate unusually large meals, and mediate stress-induced hypophagia [8,9]. Assuming that this hypophagic effects of PPG^NTS^ neurons comprises part of a wider GLP-1-mediated physiological response to acute stress [10,12], it is plausible that the tachycardic action of PPG^NTS^ neurons also specifically occurs under conditions of stress [10]. When an animal meets a stressor, rises in HR and blood pressure are induced in order to provide enough oxygen, in preparation for the fight-or-flight response [64,65]. Therefore, the tachycardia induced by activation of the PPG^NTS^ neurons could represent the role of the PPG neurons during a stress response and would explain why inhibition of the neurons does not reduce HR under resting conditions. In support of this, Ghosal et al. demonstrated that hypothalamic GLP-1Rs contribute to stress-induced tachycardia [13] and the recent whole-brain mapping of monosynaptic inputs to PPG^NTS^ neurons revealed dense innervation from stress-responsive brain regions involved in autonomic control [66]. Future investigations should focus on the necessity for PPG neuron activity, and in particular the PPG^NTS^→CAA/IML pathway, in the cardiovascular and hypophagic responses to stress.

## 5 Conclusion

In this study, we confirm the sympathoexcitatory effects of GLP-1R stimulation and show for the first time that direct application of GLP-1 to the spinal cord is sufficient to elicit tachycardic responses. We also demonstrate that, while GLP-1-producing PPG^NTS^ neurons do not provide a tonic sympathetic drive to the heart and are not necessary for the tachycardic effects of systemic Ex-4, they do have the ability to increase HR in mice. This suggests that under certain physiological conditions, PPG neurons may lead to sympathoexcitation, potentially by triggering release of GLP-1 in the spinal cord, resulting in an increase in chronotropic sympathetic drive to the heart. These physiological conditions are likely to include stress, which is known to increase sympathetic nervous system activity and have been shown to activate PPG neurons [8,9,21]. Our findings reveal a potential novel role for GLP-1 and PPG neurons in cardiovascular control through the activation of spinal cord neurons.

## Author contributions

The project was conceived by ST and AVG. Data generation was led by MKH, NM and DRC with the assistance of DIB, JER and ST. FR provided mouse lines. The manuscript was drafted by MKH and ST with input from all other authors.
