# Supplementary material for: PPG neurons in the nucleus of the solitary tract modulate heart rate but do not mediate GLP-1 receptor agonist-induced tachycardia in mice
Source: Mol Metab. 2020 May 21;39:101024. doi: 10.1016/j.molmet.2020.101024 (PMC7317700; doi:10.1016/j.molmet.2020.101024)
Supplement: Multimedia component 1 [file mmc1.docx]

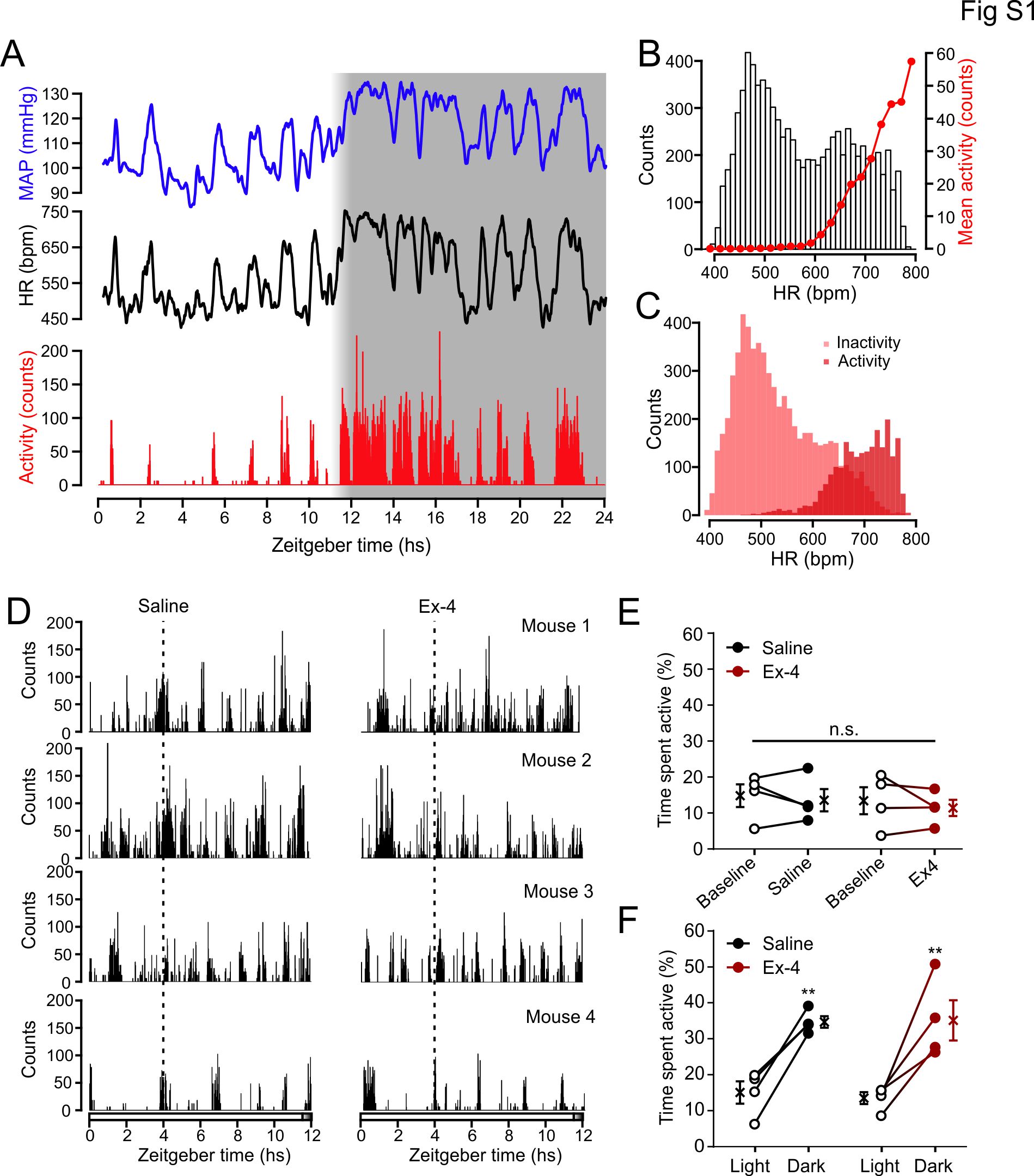


**Figure S1: PPG^NTS^ neurons are not necessary for the tachycardic response to systemic GLP-1R activation**

A) MAP (blue, top), HR (black, middle) and locomotor activity (red, bottom) measured from a single, naïve mouse over 24 h as indicated in zeitgeber time. Dark phase is indicated with a grey box with the gradient indicating a half hour “twilight” period in which lights were dimmed. Traces are running averages over 20 min. B) Histogram displaying the bimodal distribution of HR values in 10 bpm bins recorded from a single mouse over 24 h. Overlaid in red are the mean activity counts at the HR levels indicated on the x-axis. At activity level 0, HR ranged between 390 and 570 bpm. At higher mean activity levels higher HR was associated with higher activity. C) The distribution of HR values from B) split into two histograms according to activity levels with the distribution of HR values at inactivity (when activity=0) displayed in light red, and the distribution of HR values during active times in dark red. Importantly, neither distribution was normal with inactive HR values skewed to the right and active HR values skewed to the left. For this reason, estimates of resting and active HR and MAP values for individual mice implanted with biotelemetry probes are all based on the median. D) Activity levels of naïve mice (n=4) implanted with biotelemetry probes and injected i.p. with saline (left) or 10 µg/kg Ex-4 (right) 4 h into light phase. Activity levels were monitored over 12 h during light (white bar) and twilight phase (gradient bar). Zeitgeber time is indicated at the bottom. Times of i.p. injections with either saline or Ex-4 are indicated with dotted lines. E) Percentage time spent active before (Baseline, 1-4 h, open circles) and after (Saline/Ex-4, 5-8 h, filled circles) i.p. injection of saline (black) or 10 µg/kg Ex-4 (red) 4 hours into light phase. Drug x time: *F*_(1, 3)_ = 0.034, p=0.87; main effect of time (p=0.29) and drug (p=0.39). F) Percentage time spent active during light (open circles, 8-11 h) and dark phase (filled circles, 12-15 h) following injection of Ex-4 (10 µg/kg) 4 h into light phase. Drug x time: *F*_(1, 3)_ = 0.096, p=0.78; no main effect of drug (p=0.83), but a significant main effect of time (**p=0.0058).


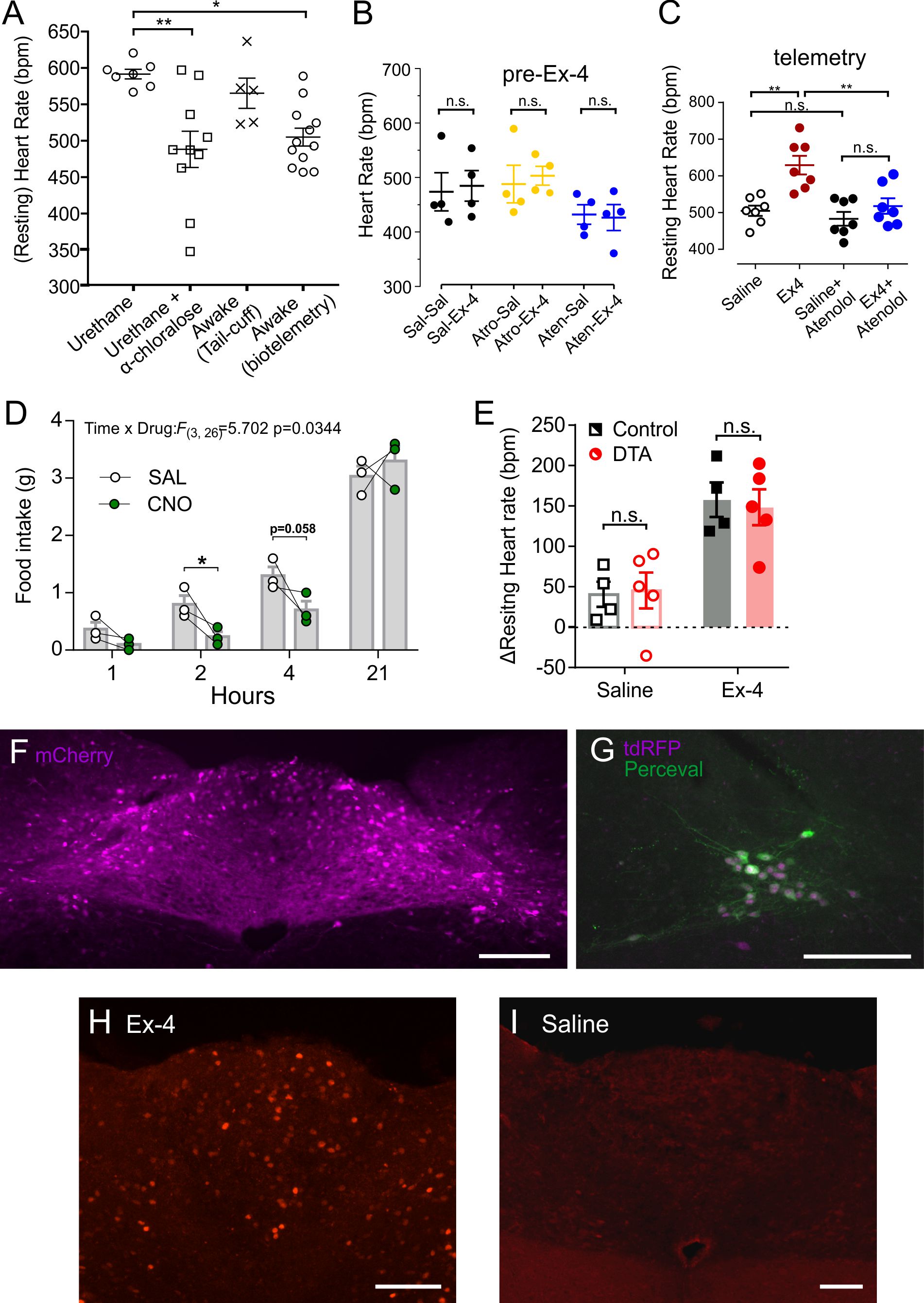


**Figure S2:**

A) Resting HR under two types of anaesthesia and in awake animals using either tail-cuff measurements with restraint or biotelemetry blood pressure probes in freely behaving mice. One-way ANOVA: *F*_(3, 31)_ = 5.812, p=0.0028 followed by Tukey’s post-hoc tests (*p<0.05, **p<0.01) B) HR in mice injected i.p. with saline, atropine (2 mg/kg), or atenolol (2 mg/kg) prior to injection with either saline or Ex-4 as indicated. Unpaired t-test revealed no significant differences as indicated by n.s. C) HR in freely behaving mice injected i.p. with Ex-4 (10 µg/kg) or saline in the absence or presence of atenolol (2 mg/kg). n=7. D) Cumulative food intake of mice expressing hM3Dq in PPG^NTS^ neurons selectively during the dark phase following i.p. injection of CNO (2 mg/kg, n=3). Drug x time: *F*_(3, 6)_ = 5.702, p=0.034; *p<0.05 according to Sidak’s multiple comparisons test. E) Change in resting HR of control (black squares) and PPG^NTS^-DTA mice (red circles) in response to saline (open symbols) and 10 µg/kg Ex-4 (filled symbols). Treatment x virus *F*_(1, 7)_ = 0.3287, p=0.5844; no main effect of virus (p=0.7610), but a significant main effect of treatment (p=0.0008). F) Representative image of mCherry (magenta) fluorescence expressed cre-independently from AAV8-mCherry-FLEX-DTA in the caudal NTS indicating spread of virus after stereotaxic injection. Scale bar: 200 μm G) Representative image of immunofluorescence double-labelling of tdRFP (magenta, indicating PPG^NTS^ neurons) and Perceval (green) in the caudal NTS demonstrating successful transduction by control AAV. Scale bar: 200 μm. H, I) Intraperitoneal injection of Ex-4 (10 μg/kg; F), but not saline (G) induced C-Fos-IR in the area postrema. Scale bars: 100 µm.
